# VTA Projection Neurons Releasing GABA and Glutamate in the Dentate Gyrus

**DOI:** 10.1523/ENEURO.0137-16.2016

**Published:** 2016-09-13

**Authors:** Niels R. Ntamati, Christian Lüscher

**Affiliations:** 1 Department of Basic Neurosciences, Medical Faculty, University of Geneva, CH-1211 Geneva, Switzerland; 2 Department of Clinical Neurosciences, Geneva University Hospital, CH-1211 Geneva, Switzerland

**Keywords:** corelease, dentate gyrus, GABA, glutamate, VTA

## Abstract

Both dopamine and nondopamine neurons from the ventral tegmental area (VTA) project to a variety of brain regions. Here we examine nondopaminergic neurons in the mouse VTA that send long-range projections to the hippocampus. Using a combination of retrograde tracers, optogenetic tools, and electrophysiological recordings, we show that VTA GABAergic axons make synaptic contacts in the granule cell layer of the dentate gyrus, where we can elicit small postsynaptic currents. Surprisingly, the currents displayed a partial sensitivity to both bicuculline and NBQX, suggesting that these mesohippocampal neurons corelease both GABA and glutamate. Finally, we show that this projection is functional *in vivo* and its stimulation reduces granule cell-firing rates under anesthesia. Altogether, the present results describe a novel connection between GABA and glutamate coreleasing of cells of the VTA and the dentate gyrus. This connection could be relevant for a variety of functions, including reward-related memory and neurogenesis.

## Significance Statement

The present studies uncover a projection from ventral tegmental area (VTA) neurons that release both GABA and glutamate to the granule cells of the dentate gyrus. The VTA is important for reward-related behaviors, while the dentate gyrus (DG) has been implicated in discriminative memory functions. Revealing the identity of the connection of the VTA to the DG thus opens avenues of investigation into how reward-related circuits might modulate memory-related circuits.

## Introduction

The mammalian reward system originates in the ventral tegmental area (VTA), from which cells project to a variety of brain regions, including the hippocampus. The hippocampus and its inputs have been implicated in memory formation, including reward-related memory (Scoville and Milner, 1957; Hernández-Rabaza et al., 2008). The VTA is made up of a heterogeneous population of dopamine (DA)-, GABA-, and glutamate (Glu)-releasing neurons (Swanson, 1982; Van Bockstaele and Pickel, 1995; Chuhma et al., 2004; Fields et al., 2007). DA neurons are the most abundant, while GABA and Glu neurons account for 35% and 2–5% of the total population, respectively (Nair-Roberts et al., 2008; Taylor et al., 2014).

All three types of VTA neurons send long-range projections. In addition to mediating local VTA inhibition that drives behavioral aversion (Tan et al., 2012; van Zessen et al., 2012), VTA GABA neurons also project to cholinergic interneurons in the nucleus accumbens (NAc) that modulate associative learning (Brown et al., 2012). VTA Glu neurons play a role in behavioral aversion through their projections to the lateral habenula (LHb) and to the NAc, where they can excite inhibitory interneurons (Root et al., 2014a; Qi et al., 2016).

The proportion of DA and non-DA axons within a given VTA projection varies by target. For instance, VTA projections to the NAc are 85% dopaminergic, while VTA projections to the hippocampus are only 6–18% dopaminergic (Swanson, 1982; Gasbarri et al., 1994). While the VTA projection to the NAc has been well characterized, studies focused on VTA projections to the hippocampus have until recently distinguished between DA and non-DA projection neurons and have relied exclusively on anatomical tracing methods. An increasing number of studies has now started focusing on this mesohippocampal pathway, but the synaptic properties of these projections have only been characterized for VTA DA neurons (Rocchetti et al., 2015; Rosen et al., 2015). Because the most abundant non-DA cell types in the VTA are GABA and Glu neurons, we hypothesized that these neurons might constitute an additional functional connection to the hippocampus.

Recent studies report that some VTA neurons corelease multiple neurotransmitters, blurring the boundaries of their neurochemical classifications. For instance, VTA DA neurons that project to the NAc and dorsal striatum can corelease DA and GABA or DA and Glu from the same axons (Chuhma et al., 2004; Stuber et al., 2010; Tritsch et al., 2012, 2014; Kim et al., 2015). Interestingly, VTA neurons that project to the LHb have been shown to corelease GABA and glutamate, thus sending mixed inhibitory/excitatory signals (Root et al., 2014b). Using a mixture of tracing methods and optogenetically assisted electrophysiological recordings, we describe a similar functional connection in which VTA neurons corelease both GABA and glutamate in the hippocampal dentate gyrus (DG). A more complete understanding of the nature of VTA projections to the hippocampus can lead to a more complete understanding of how reward-related memories are regulated.

## Materials and Methods

All animal procedures were performed in accordance with the regulations of the University of Geneva Animal Care Committee.

### Subjects

Experiments were performed on GAD65-Cre mice (Kätzel et al., 2011) and VGLUT2-Cre (Borgius et al., 2010) mice of both sexes. Genetic selectivity of Cre-recombinase expression was previously described via immunohistochemistry or *in situ* hybridization in GAD65-Cre and VGLUT2-Cre mice, respectively (Tan et al., 2012; Root et al., 2014a).

### Injection procedures

Bilateral intracranial injections of AAV5-DIO-EYFP, AAV5-DIO-ChR2(H134R)-EYFP (UNC Vector Core Facility, Chapel Hill, NC) or Alexa Fluor 555-conjugated cholera toxin subunit B (CTB555, Life Technologies) were performed under isoflurane anesthesia (2-5%; ATTANE). The injections were performed using glass capillary pipettes connected to a microinjection pump (Narishige) at a rate of ∼100 nl/min, for a total of 350 nl (viral injections) or 100 nl (CTB555 injections). The stereotaxic coordinates used were as follows [measured from bregma (in mm)]: anteroposterior (AP) −3.4, mediolateral (ML) ±0.5, and dorsoventral (DV) −4.3 for VTA; AP −2.0, ML ±1.3, and DV −2.05 for dorsal DG (dDG). All injections were performed on 3- to 5-week-old mice and were followed by a 3-week viral incubation period before further procedures were performed (i.e., age range during experiments, 6–8 weeks).

### Electrophysiology on acute slices

Horizontal and coronal hippocampal slices (300 μm) were prepared using a vibratome in ice-cold cutting solution containing the following (in mm): NaCl 87, NaHCO_3_ 25, KCl 2.5, MgCl_2_ 7, NaH_2_PO_4_ 1.25, CaCl_2_ 0.5, glucose 25, and sucrose 75. Slices were incubated in the same solution for 20 min at 31°C before being transferred to regular room temperature artificial CSF (aCSF), containing the following (in mM): NaCl 119, NaHCO_3_ 26.2, KCl 2.5, MgCl_2_ 1.3, NaH_2_PO_4_ 1, CaCl_2_ 2.5, and glucose 11. After at least 1 h for recovery, slices were transferred to the recording chamber, superfused with aCSF at 2 ml/min. All solutions were constantly bubbled with 95% O_2_ and 5% CO_2_. Bicuculline (Bic; 10 μm; Sigma-Aldrich) and NBQX (10 μm; Sigma-Aldrich) were added to the aCSF in order to block GABA_A_ and AMPA/kainate currents, respectively. Neurons were visually identified using an IR CCD camera mounted on an Olympus BX45 Microscope. Borosilicate glass pipettes at a resistance range of 2–4 MΩ were used for recording. The internal solution used contained the following (in mm): K-gluconate 30, KCl 100, MgCl_2_ 4, creatine phosphate 10, Na_2_ATP 3.4, Na_3_GTP 0.1, EGTA 1.1, and HEPES 5. The calculated reversal potential with this internal solution for Cl^−^ was −5 mV, and cells were voltage clamped at −70 mV. Currents were amplified, filtered at 2 kHz, digitized at 10 kHz, and saved on a hard disk. The liquid junction potential was small (−4 mV), and traces were therefore not corrected. Access resistance was monitored by a hyperpolarizing step of −4 mV at the onset of every sweep, and the experiment was discarded if the access resistance changed by >20%. ChR2 was stimulated with brief (4 ms) blue light pulses using a 470 nm LED mounted on the microscope and powered by an LED driver under computer control. Paired pulses were given at varying interpulse intervals (10–200 ms), and the paired-pulse ratio (PPR) was calculated as the ratio of the second postsynaptic current (PSC) over the first PSC. A threshold of three times the SD of baseline noise amplitude was used to distinguish small PSCs from noise. Representative traces were made from averaging at least 30 consecutive sweeps.

### Extracellular recordings

Mice were anesthetized with isoflurane (1–5%) and positioned on a stereotaxic frame, where a craniotomy was performed over the left VTA and dDG, and body temperature was maintained throughout the experiment with a homeothermic heating pad. A fiber optic cannula was implanted above the VTA [coordinates from bregma (in mm): AP −3.4, ML +0.45, DV −4.05 (5° angle)], secured with dental cement, and connected to a 473 nm solid-state laser (10–15 mW out of the fiber optic) for optical excitation of ChR2-expressing neurons. Laser stimulation consisted of 20 pulses (5 ms) delivered at 20 Hz. An AgCl-coated silver wire inserted in a borosilicate glass pipette (1–2 μm tip diameter) filled with 0.9% NaCl was used as the recording electrode. The reference electrode was inserted subcutaneously on the back of the mouse. In order to access the dorsal DG and the VTA, two craniotomies (4 mm^2^), centered on the injection coordinates described above were performed on the hemisphere opposite to the fiber optic implantation. Following stereotaxic coordinates, the recording electrode was slowly lowered into the DG (1.8–2.2 mm deep from the pia) or the VTA (4.0–4.5 mm deep from the pia) until a stable signal was detected. When possible, the electrode location was verified with the small tissue damage left in the DG, as assessed by DAPI staining. Electrical signals were AC coupled, amplified, and monitored in real time using a digital oscilloscope and audiomonitor, then they were digitized at 20 kHz (for waveform analysis) or 5 kHz and stored on hard disk using a custom-made program within IGOR (WaveMetrics). The bandpass filter was set between 0.3 and 2 kHz. Processed data were displayed as peristimulus time histograms (PSTHs), event raster plots, and binned firing rate plots. Time windows of 500 ms preceding (baseline), during (stimulation), or following (poststimulation) the light stimulation were analyzed to determine whether or not the recorded neuron was affected. The number of events in the three periods was compared throughout recording trials using the Wilcoxon signed-rank test.

### Histological procedures and imaging

Mice were deeply anesthetized with pentobarbital (300 mg/kg, i.p.) and transcardially perfused with 0.1 m PBS followed by 4% paraformaldehyde (PFA; Sigma-Aldrich). Brains were removed, postfixed in 4% PFA for 24 h at 4°C, and cut in 50 μm sections on a vibratome. For immunohistochemistry, slices were incubated for 1 h at room temperature in a blocking solution containing 5% bovine serum albumin (Sigma-Aldrich) and 0.3% Triton X-100 (Axon Lab AG) in 0.1 m PBS, followed by an overnight incubation at 4°C with a rabbit anti-tyrosine hydroxylase (TH) antibody (1:500; Millipore) in blocking solution. Slices were then rinsed in PBS and incubated for 2 h at room temperature with a goat anti-rabbit antibody (1:500; Invitrogen) in blocking solution, then rinsed in PBS again. Slices were then mounted on glass slides with Fluoroshield Mounting Medium with DAPI (Abcam). For the visualization of biocytin-filled neurons from *ex vivo* whole-cell recordings, slices were fixed in 4% PFA for 2 h at room temperature and incubated overnight in blocking solution containing Cy3-streptavidin (1:1000; Invitrogen). Slices were then mounted as above. Confocal images were captured with a Zeiss LSM 510 Meta Laser Scanning Microscope or a Nikon A1r Spectral Scanning Confocal Microscope, and then processed with Zeiss LSM Image Browser and ImageJ software.

### Data analysis and statistics

Data were analyzed with Igor, Microsoft Excel, and Prism software, and are expressed as the mean ± standard error of the mean (SEM). Statistical analysis was performed using the Mann–Whitney test, the Kruskal-Wallis test, or the Friedman test with Dunn’s *post hoc* test.

## Results

### The hippocampus receives GAD65^+^ afferents from the midbrain

**Figure 1.**
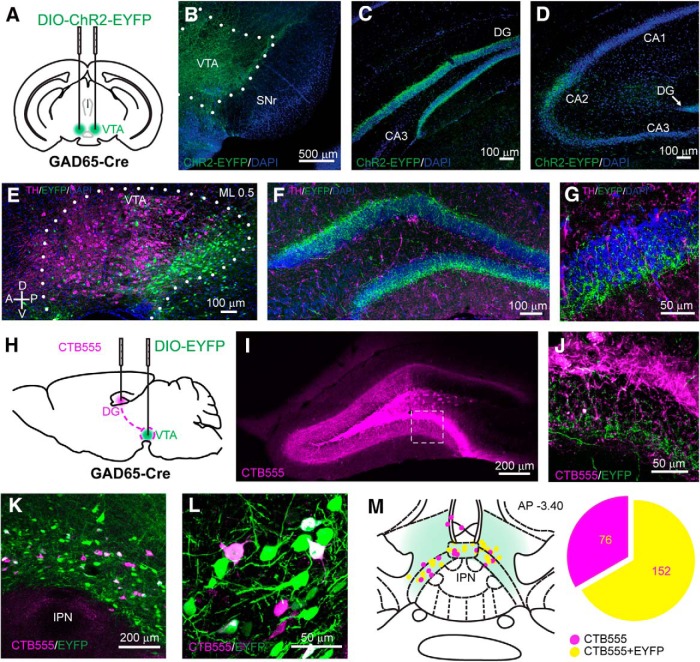
VTA GABA cells project to the hippocampus. ***A***, Schematic of the viral injection of the Cre-dependent ChR2-EYFP construct in the VTA of GAD65-Cre mice. ***B***, Coronal section of the VTA showing the expression of ChR2-EYFP in GABA neurons. ***C***, ***D***, ChR2-EYFP-expressing axons are observable in the GCL of the DG (***C***) and in the CA2 region (***D***). ***E***, Sagittal section of illustrating the anteroposterior distribution of the infected GABA neurons withing the VTA. The ML coordinate from the midline is indicated at the top. ***F***, ***G***, Confocal images at low (***F***) and high (***G***) magnification showing the absence of colocalization between VTA GABA fibers and TH in the DG. ***H***, Schematic showing the injection of CTB555 into the dorsal DG and of a DIO-EYFP-expressing viral vector into the VTA of a GAD65-Cre mouse. ***I***, Coronal section of the dDG showing the CTB555 injection site. ***J***, Higher-magnification image detailing the presence of EYFP-expressing axons in the GCL. ***K***, ***L***, Confocal images at low (***K***) and high (***L***) magnification showing the retrograde labeling of CTB555 in medial VTA neurons, some of which are EYFP-expressing GABA neurons. ***M***, Schematic representation of the localization of the retrogradely labeled neurons within the VTA and the proportion of neurons colocalizing with EYFP. The AP coordinate from bregma is indicated at the top.

Because GABA neurons make up ∼35% of neurons in the VTA, they likely contribute to the non-DA innervation of the hippocampus (Gasbarri et al., 1994). We therefore started with anterograde tracing of VTA GABA axons, using transgenic mice expressing Cre recombinase under the promoter for the *Gad2* gene (GAD65-Cre; Kätzel et al., 2011). We stereotaxically injected an adeno-associated viral vector expressing a double-floxed inverted open reading frame encoding enhanced yellow fluorescent protein (EYFP) fused with channelrhodopsin (DIO-ChR2-EYFP; the latter with the goal to functionally probe connectivity, as described below) bilaterally into the VTA (Fig. 1*A*). This enabled us to selectively target GAD65^+^ neurons within the VTA with minimal ChR2-EYFP expression in the adjacent GABA-rich SNr (Fig. 1*B*) and no nonspecific expression in VTA DA neurons (Tan et al., 2012). We observed the presence of ChR2-EYFP-expressing fibers in the hippocampus and found the highest fiber density in the granule cell layer (GCL) of the DG (Fig. 1*C*). No ChR2-EYFP-expressing fibers were found in the CA3 or the CA1, and only sparse fibers were detected in the CA2 (Fig. 1*D*). To confirm the nondopaminergic identity of these projections, we immunostained for TH, a marker of DA-producing neurons (Fig. 1*E*). While sparse TH-positive fibers could be observed in the DG, we did not find any colocalization with the EYFP-expressing VTA axon terminals (Fig. 1*F*,*G*). These anterograde tracing experiments suggest that GABAergic mesohippocampal neurons send their axons preferentially to the DG.

We then sought to confirm this result with retrograde labeling. To this end, we transduced the VTA of GAD65-Cre mice with a floxed EYFP and injected the retrograde tracer cholera toxin subunit B conjugated to a red fluorophore (CTB555) into the dDG (Fig. 1*H*). Three weeks after these injections, the CTB555 injection location in the DG was still evident (Fig. 1*I*), and, at high magnification, we could observe EYFP-expressing fibers lining the GCL (Fig. 1*J*). We found neurons in the VTA that were positive for both EYFP and CTB555, confirming the existence of midbrain GABAergic projections to the DG (Fig. 1*K*,*L*). The DG-projecting neurons (i.e., CTB positive) were confined to a narrow band along the anteroposterior axis in the ventromedial VTA, just above the interpeduncular nucleus, and 66.7% of these cells expressed GAD65. These data suggest that the majority of VTA afferents to the DG are GABAergic (Fig. 1*M*).

### Optogenetic probing reveals a functional connection

**Figure 2.**
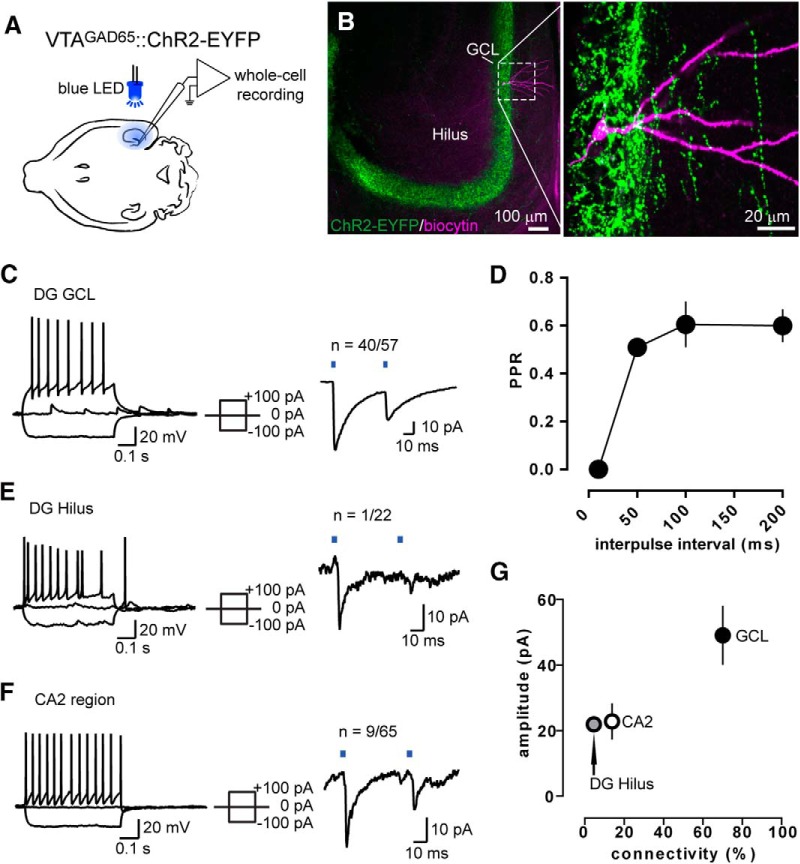
DG granule cells receive input from VTA GABA axons. ***A***, Schematic for the whole-cell patch-clamp experiments. ***B***, Sample neuron loaded with biocytin after recording in the GCL. ***C***, Left, Example trace of the current-clamp responses to current steps. Right, Example voltage-clamp trace in a paired-pulse light stimulation protocol. ***D***, PPR of the light-evoked currents in GCL neurons measured at different interpulse intervals. ***E***, Same as in ***C*** but for neurons recorded in the hilus. ***F***, Same as in ***C*** and ***E***, but for CA2 neurons. ***G***, Mean amplitude (±SEM) of the light-evoked currents in the GCL, hilus, and CA2 plotted against the percentage of connected neurons.

To confirm that these VTA GABA neurons are functionally connected to DG neurons, we used optogenetic tools and brain slice electrophysiology. We again expressed ChR2 in the GABA neurons of the VTA and then took slices of the hippocampus. We performed whole-cell recordings in DG neurons and optogenetically evoked PSCs by exposing the slice to wide-field illumination with a blue LED (Fig. 2*A*). Since most of the ChR2-expressing fibers were located in the GCL (Figs. 1*C*, 2*B*), we first recorded from granule cells. We were able to evoke small PSCs (40.3 ± 5.3 pA) with a short onset latency (3.0 ± 0.1 ms) in most of these cells (*n* = 40 of 57). We also measured the PPR and found a strong paired-pulse depression, suggesting a high initial release probability at these synapses (Fig. 2*C*,*D*). We then investigated whether light-responsive and nonresponsive neurons had different basic electrophysiological properties, and found that resting membrane potential, capacitance, input resistance, and input–output responses were similar between groups (Tables 1, 2).

**Table 1:** Basic electrophysiological properties in light-responsive and nonresponsive DG granule cells

	Light-responsive neurons (*n* = 15)	Nonresponsive neurons (*n* = 15)	p Value (light-responsive vs nonresponsive)
Resting membrane potential	−72.9 ± 1.2 mV	−70.8 ± 2.1 mV	0.5606
Capacitance	14.2 ± 1.4 pF	13.6 ± 1.4 pF	0.8519
Input resistance	232.5 ± 24.5 MΩ	253.4 ± 25.8 MΩ	0.5336

Quantification of resting membrane potential, capacitance, and input resistance values shows no difference between DG granule cells receiving the VTA GABA input and nonconnected neurons (i.e. light-responsive vs nonresponsive neurons).

**Table 2:** Statistics

Figure	Panel	Data structure	Type of test	*p* Value
2	G	Non-normal distribution	Kruskal–Wallis test	0.2086
3	C	Non-normal distribution	Mann–Whitney test	0.0013
	D	Non-normal distribution	Mann–Whitney test (time to peak)	0.0102
		Non-normal distribution	Mann–Whitney test (decay)	0.0004
		Non-normal distribution	Mann–Whitney test (onset)	0.2817
		Non-normal distribution	Mann–Whitney test (PPR)	0.6048
4	E	Non-normal distribution	Mann–Whitney test	0.1921
	H	Non-normal distribution	Mann–Whitney test	0.0013
	J	Non-normal distribution	Mann–Whitney test (time to peak)	0.0045
		Non-normal distribution	Mann–Whitney test (decay)	0.0006
		Non-normal distribution	Mann–Whitney test (onset)	0.2814
		Non-normal distribution	Mann–Whitney test (PPR)	0.3020
5	C	Non-normal distribution	Friedman test	Friedman statistic: 15.80; <0.0001
			Dunn’s multiple comparison post-test (baseline vs stimulation)	Rank sum difference: 13.00; 0.0110
			Dunn’s multiple comparison post-test (baseline vs poststimulation)	Difference in rank sum: -4.000; >0.9999
			Dunn’s multiple comparison post-test (stimulation vs poststimulation)	Difference in rank sum: -17.00; 0.0004
Table 1		Non-normal distribution	Mann–Whitney test (resting membrane potential)	0.5606
		Non-normal distribution	Mann–Whitney test (capacitance)	0.8519
		Non-normal distribution	Mann–Whitney test (input resistance)	0.5336

The GCL also contains the apical dendrites of a variety of DG interneurons residing in the hilus and hilus/GCL border (Freund and Buzsáki, 1996; Amaral et al., 2007). We therefore recorded from hilar interneurons and found that only 1 of 22 neurons was light responsive, with a rather small IPSC amplitude (21.9 pA; Fig. 2*E*). We finally probed for light-evoked responses in the CA2 region, where we had also observed some VTA GABA fibers (Fig. 1*D*), and we identified a few connected neurons sparsely distributed throughout the strata oriens, pyramidale, and radiatum (22.8 ± 5.3 pA, *n* = 9 of 65; Fig. 2*F*). Together, these results confirm the presence of functional synaptic connections between VTA GABA neurons and DG granule cells (Fig. 2*G*).

### GAD65^+^ mesohippocampal fibers corelease glutamate

**Figure 3.**
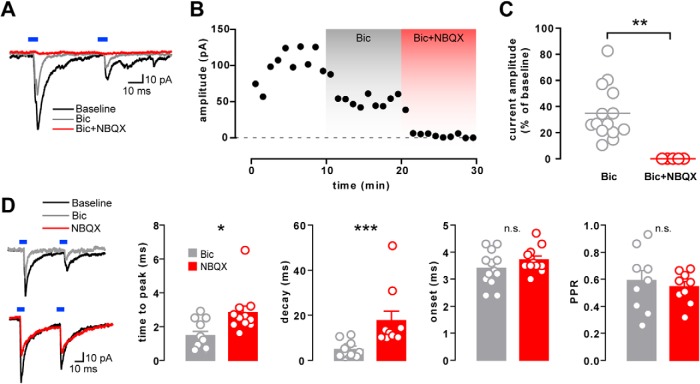
Mixed neurotransmission onto DG granule cells. ***A***, Example voltage-clamp traces of a GCL neuron before and after bath application of bicuculline alone (Bic) or in combination with NBQX (Bic+NBQX). ***B***, Time course of the current peak amplitude (1 min bins) from the same example neuron as in ***A***. ***C***, Residual current amplitude following Bic (*n* = 14) or Bic+NBQX (*n* = 5) application, shown as a percentage of the baseline current amplitude. ***D***, Sample traces before and after the application of Bic or NBQX alone and the effect on the time to peak (*n* = 11, 11), decay time (*n* = 13, 11) onset latency (*n* = 13, 10), and PPR (*n* = 9, 9) of the light-evoked currents.

In order to confirm the GABAergic nature of the light-evoked PSCs in DG granule cells, we antagonized the GABA_A_ receptor by bath applying 10 μm Bic. Surprisingly, this saturating concentration failed to abolish the currents, leaving a residual PSC (34.9 ± 5.5% of baseline current; *n* = 14; Fig. 3*A–C*). This residual PSC was blocked by adding the AMPA/kainate receptor antagonist NBQX to the bath (10 μm; *p* = 0.0013; *n* = 5; Fig. 3*A–C*).

AMPA-mediated currents are generally faster than GABA_A_-mediated currents (Hestrin et al., 1990; Banks et al., 1998). To test whether this is also true for the dual PSC observed in the DG, we pharmacologically isolated the Bic- or NBQX-resistant components of the currents (Fig. 3*D*). Bic-resistant currents (putative Glu PSCs) had shorter time to peak (1.5 ± 0.3 vs 2.8 ± 0.4 ms; *p* = 0.0102; *n* = 11, 11) and shorter decay time (4.8 ± 0.9 vs 17.7 ± 4.2 ms; *p* = 0.0004; *n* = 13, 11) compared with the NBQX-resistant component (putative GABA PSCs). Neither Bic nor NBQX affected the onset latency of the light-evoked currents (Bic vs NBQX: 3.4 ± 0.2 vs 3.7 ± 0.1 ms; *p* = 0.2817; *n* = 13,10) or PPR (Bic vs NBQX: 0.6 ± 0.1 vs 0.5 ± 0.0; *p* = 0.6048; *n* = 9, 9). Together, these findings strongly suggest dual monosynaptic GABA and glutamate transmission that arises from the same VTA GAD65^+^ axons.

### VGLUT2^+^ VTA neurons project to the DG and corelease GABA

**Figure 4.**
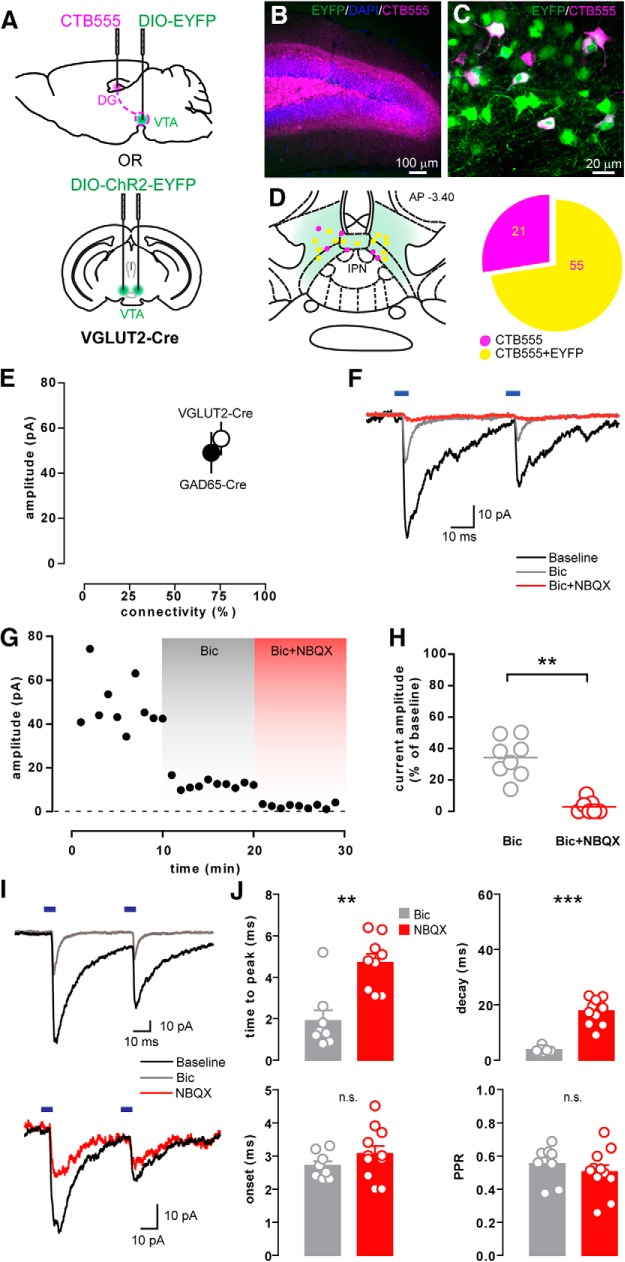
VTA glutamatergic neurons release GABA in the DG. ***A***, Schematic showing the injection procedures for retrograde tracing or for whole-cell recordings in VGLUT2-Cre mice (as done in Fig. 1*A*,*H* for GAD65-Cre mice). ***B***, CTB555 injection site in the dDG. ***C***, High-magnification image of the retrogradely labeled neurons in the VTA, some of which colocalize with EYFP. ***D***, Schematic localization of retrogradelylabeled cells and the proportion of CTB-positive neurons colocalizing with EYFP in VGLUT2-Cre mice. ***E***, Graph showing the average amplitude (±SEM) of the light-evoked PSCs recorded in the GCL of VGLUT2- or GAD65-Cre mice as a function of their connectivity. ***F***, Example traces showing the light-evoked PSC recorded in a granule cell at baseline and after bicuculline alone (Bic) or together with NBQX (Bic+NBQX). ***G***, Peak amplitude time course from the same example neuron in ***F***. ***H***, Residual current amplitude after Bic (*n* = 8) or Bic+NBQX (*n* = 7) application, shown as a percentage of the baseline current amplitude. ***I***, Sample traces before and after the application of Bic or NBQX alone. ***J***, Effect of Bic or NBQX alone on the time to peak (*n* = 8,9), decay time (*n* = 7,11), onset latency (*n* = 8,11), and PPR (*n* = 8,11) of the light-evoked currents.

Because these results suggest the existence of DG-projecting VTA GABA neurons that corelease glutamate, we also tested whether VTA glutamate neurons might corelease GABA in the DG. To target glutamate neurons, we used mice expressing Cre recombinase under the promoter for the *Slc17a6* gene (VGLUT2-Cre). We then injected CTB555 into the dDG to visualize neurons in the VTA that project to the DG and transfected the VTA with floxed EYFP to label the VTA glutamate neurons (Fig. 4*A*). We found that 72.4% of all CTB-positive VTA neurons were colabeled with EYFP (Fig. 4*B–D*), suggesting that the majority of DG-projecting neurons in the VTA are glutamatergic. Notably, the percentage of DG-projecting neurons in the VTA that are GABAergic is similar to the proportion of neurons that are glutamatergic. A separate group of VGLUT2-Cre mice was transfected with floxed ChR2-EYFP in the VTA for patch-clamp experiments aimed at functionally characterizing these VTA Glu projections to the DG (Fig. 4*A*). Whole-cell recordings of individual DG granule cells were performed in hippocampal slices, and PSCs were evoked with blue LED exposure, as described before. We identified mesohippocampal synaptic currents that were partly inhibited by Bic (34.3 ± 4.4% of baseline; *n* = 8) and further blocked by coapplication of NBQX (2.9 ± 1.6% of baseline; *n* = 7; *p* = 0.0013; Fig. 4*F–H*), similar to the synaptic currents evoked from VTA GABA neurons in the DG. The average PSC amplitude (55.3 ± 7.0 pA; *n* = 31), the percentage of connected cells (75.6%; Fig. 4*E*), and the modulation of current kinetics by Bic and NBQX (Fig. 4*I*,*J*) were all similar to those observed in GAD65-Cre mice. These results confirm the presence of a population of VTA neurons that release both GABA and glutamate in the mouse DG, and indicate that the majority of VTA neurons that project to the DG contain both GABA and glutamate.

### VTA optogenetic stimulation *in vivo* decreases DG firing

**Figure 5.**
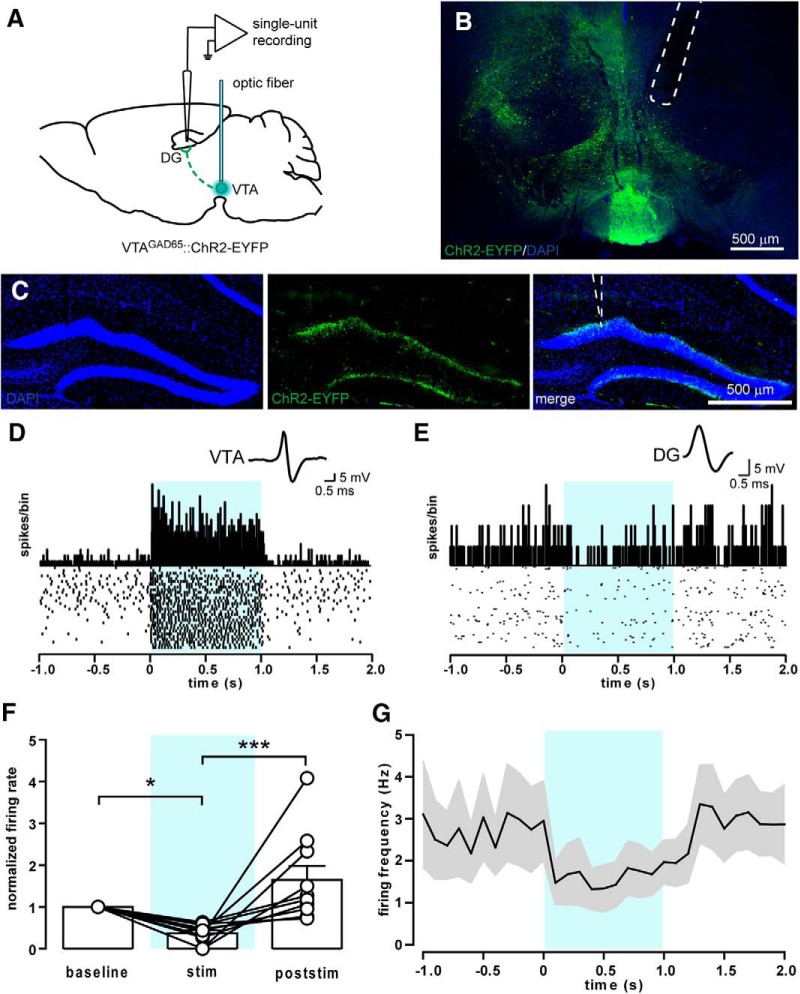
Stimulation of VTA GABA neurons reduces firing activity in the DG *in vivo*. ***A***, Schematic of the single-unit recordings in the DG of anesthetized mice. An optic fiber connected to a blue laser was implanted above the VTA for the light stimulation of ChR2-expressing GABA neurons (20 5 ms pulses at 20 Hz). ***B***, Sample confocal image showing the optical fiber positioning above the VTA. ***C***, Sample confocal images showing the position of the recording electrode in the upper blade of the DG. ***D***, Spike waveform, PSTH, and raster plot of a sample GABA neuron recorded in the VTA and showing an increase in firing rate upon blue light stimulation (light blue-shaded area). ***E***, Spike waveform, PSTH, and raster plot of a sample responsive neuron in the DG showing a moderate reduction in the firing rate during blue light stimulation. ***F***, Average firing rate of responsive neurons during (stim) or after (poststim) light stimulation normalized to baseline firing (*n* = 10). ***G***, Average firing frequency of responsive neurons recorded in the DG (*n* = 10).

Given that non-DA VTA neurons release both excitatory and inhibitory neurotransmitters in the DG, we investigated how the activation of these neurons modulates the activity of DG neurons *in vivo*. We performed single-unit extracellular recordings in anesthetized GAD65-Cre mice expressing floxed ChR2 in the VTA. Blue light was delivered to the medial VTA via an optic fiber connected to a blue laser (Fig. 5*A–B*), while the recording electrode was lowered into the dorsal DG (Fig. 5*C*). VTA neurons were light stimulated for 1 s at 20 Hz, a frequency shown to reliably excite VTA GABA neurons (Fig. 5*D*; Brown et al., 2012; Tan et al., 2012). We identified 10 units in the DG that significantly changed their firing rates during optogenetic stimulation compared with periods immediately before (baseline) and after stimulation (of 25 that we were able to isolate; Fig. 5*E*). All 10 stimulation-responsive units were inhibited by light delivery in the VTA (62.7 ± 7.2% inhibition of spiking activity; Fig. 5*E–G*). We conclude that the mesohippocampal connection is functional *in vivo* and that the net effect of its mixed GABA/glutamate transmission is inhibition.

## Discussion

We characterize a population of non-DA neurons in the VTA that project to the DG. These neurons corelease GABA and glutamate, and can be targeted with either GAD65-Cre or VGLUT2-Cre mouse lines. Given that each line labels >50% of VTA neurons that project to the DG coreleasing GABA and glutamate, a given neuron likely expresses both GAD65 and VGLUT2. We also show that the net effect of stimulating these VTA neurons is the functional inhibition of DG neurons.

While the mesohippocampal pathway has been described by a number of studies (Amaral and Cowan, 1980; Scatton et al., 1980; McNamara et al., 2014; Rosen et al., 2015; Broussard et al., 2016), most have focused solely on the DA projections, with only a few acknowledging the presence of non-DA neurons connecting the VTA or SNc with the hippocampus (Swanson, 1982; Gasbarri et al., 1994). Here we identify a previously unexamined population of non-DA neurons in the VTA that targets the DG.

A recent study has shown that the rat DG receives very little innervation from midbrain DA neurons and that virtually all TH-immunoreactive axons originate from noradrenergic neurons in the locus ceruleus (Ermine et al., 2016). These results are in accordance with our finding that the mesohippocampal terminals under study do not colocalize with TH, and are mostly GABAergic and glutamatergic. Our retrograde tracing experiments show that neurons projecting toward the dDG tend to localize near the midline of the VTA, as previously shown by other groups (Gasbarri et al., 1994; Ermine et al., 2016). However, neurons projecting to different dorsoventral segments of the DG might be located throughout the VTA, both rostrally and caudally. Our injections, covering most of the VTA, likely also include the GABA-rich tail of the VTA or rostromedial tegmental nucleus (RMTg), a structure that has recently been characterized in rats and nonhuman primates (Jhou et al., 2009; Hong et al., 2011). It therefore cannot be excluded that some RMTg neurons contribute to this mesohippocampal pathway. However, in the mouse, the boundary between the VTA and RMTg is fuzzy, which may make the labeling of caudal midbrain non-DA neurons (posterior VTA vs anterior RMTg) somewhat arbitrary.

Our study relies on Cre transgenic mouse lines to identify specific cell types and optogenetics to restrict excitation to the genetically and spatially defined synapses of interest (Lammel et al., 2015; Stuber et al., 2015). For the GAD65 and VGLUT2 Cre lines used here, the selectivity has been validated (Borgius et al., 2010; Tan et al., 2012; Root et al., 2014a). The absence of antibodies that, in our hands, reliably stain for GABAergic and glutamatergic somata makes it difficult to directly visualize and quantify the neurons synthetizing both neurotransmitters with immunohistochemical methods. Further experiments employing *in situ* hybridization techniques would be necessary for this purpose. Nonetheless, our evidence for GABA/glutamate corelease is further confirmed by the crossover observation in both of these Cre lines. These findings relate to reports of a variety of VTA neuronal populations that may corelease multiple neurotransmitters (Chuhma et al., 2004; Stuber et al., 2010; Tritsch et al., 2012; Root et al., 2014b). GABA/Glu coreleasing neurons, specifically, have been described previously in the VTA, cerebral cortex, basal ganglia, hippocampus, and brainstem auditory system (Noh et al., 2010; Beltrán and Gutiérrez, 2012; Shabel et al., 2014; Fattorini et al., 2015), suggesting that our findings likely do not stem from a flawed genetic strategy.

Our whole-cell voltage-clamp recordings showed that these VTA-to-DG synapses display a high initial release probability, as assessed by a PPR that was <1 for the range of interpulse intervals used (10–200 ms). However, viral-mediated expression of ChR2 has been reported to lead to altered short-term plasticity (Jackman et al., 2014), which will have to be kept in mind when using PPR as an indicator of release probability (e.g., characterizing synaptic plasticity).

It could be argued that AMPA and GABA_A_ receptor-mediated currents arise from collateral feedforward monosynaptic and polysynaptic connections. For example, VTA Glu neurons might synapse directly onto DG granule cells but also connect to DG interneurons. These interneurons could then mediate the GABAergic component of the light-evoked currents that we observed. However, our observations make this possibility very unlikely. The latency to the PSC onset is too short for polysynaptic Glu-GABA components. Moreover, in a feedforward scenario, NBQX would suppress both components of the current, which is not what we observed. Finally, we found that only 1 of 22 interneurons in the DG received functional input from the VTA. Together, our data strongly argue in favor of a corelease of GABA and glutamate in the DG. Nevertheless, the contribution of a fast disynaptic connection remains possible. This could be further investigated with paired recordings on DG granule cells and the few interneurons innervated by VTA axons.

Our *in vivo* recordings revealed a net inhibition of DG granule cells with VTA GABA neuron stimulation. The recordings were performed under isoflurane-induced anesthesia, which can enhance inhibitory tone and decrease spontaneous neuronal firing rates (Detsch et al., 2002). In general, in the awake animal, DG granule cells fire at frequencies <0.15 Hz, with only 9% firing >2 Hz (Neunuebel and Knierim, 2012). As a consequence, the majority of DG granule cells were likely undetectable under anesthesia, potentially biasing our recordings to the neurons with the highest spontaneous activity. It is therefore possible that in awake, freely moving animals, non-DA projection from the VTA may have additional effects.

The present study identifies and characterizes a connection from the VTA to the DG that uses both GABA and Glu as its transmitters. These neurotransmitters generally exert opposing effects, and we have found that the inhibitory component of transmission at this synapse prevails *in vivo*. Previous studies that have investigated GABA and glutamate corelease have reported that the balance between GABA release and glutamate release can be shifted in an activity-dependent manner (Fattorini et al., 2015). The shifting relative strength of GABA and glutamate transmission at mixed synapses in the LHb has been implicated in mood regulation and negative affect (Shabel et al., 2014). The balance between GABA and Glu release at the inhibitory/excitatory VTA inputs to the DG that we report might be regulated by a variety of experiences, including reward-related experiences. Given the activity dependence of DG neurogenesis (Rubio-Casillas and Fernández-Guasti, 2016) and its potential importance in drug-related pathophysiology (Castilla-Ortega et al., 2016), it is tempting to speculate that the projection from the VTA to the DG characterised here might be capable of dynamically modulating neurogenesis. This non-DA mesohippocampal projection has now been characterized and is ripe for further inquiry into its functional relevance.

## Synthesis

Pablo Castillo, Albert Einstein College of Medicine

Synthesis

The decision was a result of the Reviewing Editor Pablo Castillo and the peer reviewers coming together and discussing their recommendations until a consensus was reached. A fact-based synthesis statement explaining their decision and outlining what is needed to prepare a revision is listed below. The following reviewers agreed to reveal their identity: Stephen Rayport, Julie Kauer

Your manuscript has been evaluated by two reviewers. The following is a synthesis of the online discussion on your study. The reviewers agree on the high quality of the results and overall merit of your findings, which clearly demonstrate functional GABAergic and glutamatergic connections from the mouse VTA to the dentate gyrus. They both agree that the strength of your study is in combining the GAD-cre and VGLUT2-cre lines to identify the respective projections, combined with retrograde labeling, and physiological demonstration of function. Because your study does not formally discard that GABA/glutamatergic projections could also be dopaminergic, you may want to consider "putative non-DA neurons/pathway" throughout the manuscript. As you will see in the specific comments to the authors, the reviewers also request clarification on a number of issues, including the specificity of the projecting neurons (i.e. VTA vs non-VTA), proportion of GABA/Glu containing terminals, and age of the animals. Finally, it is expected that the revision of your manuscript requries less than 2 months' work.

REVIEWER 1:

1. The principal premise that the authors are targeting non-dopaminergic VTA neurons is not directly addressed. As GAD is colocalized in about 10-15% of dopamine neurons and VGLUT2 in as many as 50%, it is likely that many of the neurons examined are in fact also dopaminergic.

2. The study ignores the major source of VTA GABA synthesis, namely aldehyde dehydrogenase 1a1: Kim J-I, Ganesan S, Luo SX, Wu Y-W, Park E, Huang EJ et al. (2015) Aldehyde dehydrogenase 1a1 mediates a GABA synthesis pathway in midbrain dopaminergic neurons. Science, 350:102-6.

3. The relationship between GABAergic and Glutamatergic status is not addressed; what fraction of GAD-cre neurons are both GABAergic and Glutamatergic? What fraction of VGLUT2-cre neurons are both?

4. The statement (Intro, page 2) that "the synaptic properties of the projections from the VTA to the hippocampus have not been characterized." bears qualification. See for example:

a. Rosen ZB, Cheung S, Siegelbaum SA (2015) Midbrain dopamine neurons bidirectionally regulate CA3-CA1 synaptic drive. Nat Neurosci 18:1763-71.

b. Mingote S, Chuhma N, Kusnoor SV, Field B, Deutch AY, Rayport S (2015) Functional connectome analysis of dopamine neuron glutamatergic connections in forebrain regions. J Neurosci 35:16259-71.

c. Rocchetti, J., Isingrini, E., Dal Bo, G., Sagheby, S., Menegaux, A., Tronche, F., et al. (2015). Presynaptic D2 dopamine receptors control long-term depression expression and memory processes in the temporal hippocampus. Biological Psychiatry, 77, 513-25.

5. The statement (Intro, page 2) "VTA DA neurons that project to the NAc can co-release DA and GABA or DA and Glu from the same axons" Is misleading. DA neurons that corelease GABA project preferentially to the dorsal striatum, not the NAc.

6. The use of "juvenile or adult" mice spans maturation and raises concerns that the results obtained may differ based on the age of the animals. Results should therefore be parsed by animal age (juvenile vs. adult) and compared.

REVIEWER 2:

This is a very interesting manuscript using optogenetic/electrophysiological, immunohistochemical and tracing methods to elucidate the functions of projections from GABAergic VTA neurons to the dentate gyrus. The authors show convincingly that GABAergic VTA neurons labeled using the Gad2-Cre mouse project to the granule cell layer of the dentate (as well as to CA2 of hippocampus). Optogenetic activation of these fibers elicits a mixed GABAergic and glutamatergic PSC in dentate granule cells; by contrast, most hilar neurons and CA2 neurons only rarely had a PSC. Blocking GABAA receptors revealed a bicuculline-resistant fast EPSC that could be blocked by NBQX, indicating that it resulted from AMPAR activation. Given the apparently inhibitory/excitatory effects of these afferents, they then performed in-vivo recordings, and found net inhibition of firing of dentate granule cells. The manuscript is nicely done, and uses cutting edge tools to define a connection previously studied only using anatomical tracing methods.

Suggestions for revision:

1. One difficulty with studying VTA GABA neurons is isolating their effects from those of the neighboring GABAergic cells in the RMTg. The authors should include images indicating the anterior-posterior spread of virus in VTA injected animals, and discuss the possibility of RMTg neurons being included in the study. The injection coordinates reported in the methods are used in other labs to target the RMTg, a region well-known to have GABAergic neurons.

2. PPR data should be interpreted cautiously, as viral-mediated expression of ChR2 constructs has been shown to artificially raise the release probability (Jackman et al, Journal of Neuroscience, 2014)

3. There are many interneurons in the dentate gyrus that could be innervated by the VTA neurons other than those in the hilus. The authors may want to record from a few interneuron somata in the molecular layer to be sure that these do not receive direct excitatory input from the VTA cells and then disynaptically inhibit the granule cells. The authors argue against a disynaptic inhibition based on the latency of the input, but the light pulses are relatively long, leaving this as a possibility when no synaptic blockers are present. The evoked response in NBQX will lack any polysynaptic inputs, but does not rule out a disynaptic effect that could contribute to the inhibition shown in vivo or when no blockers are present.

4. Are the results not also consistent with PSCs resulting from the activation of multiple terminals/neurons that release either GABA or glutamate?

5. While it is not essential, and perhaps the authors have another manuscript in process, it would be of great interest in the field to know whether the authors also found any glutamatergic or GABAergic inputs from dopamine neurons the VTA when they labeled these with ChR2.

Minor:

1. What was the age range for surgeries? What was the age range for data collection? Three week old mice are not adults, they are just beginning adolescence, and there is significant development continuing during this period in the VTA and dentate gyrus.

2. Figure 5: There is a discrepancy between the figure legend and methods section as to whether light pulses are 10 ms or 5 ms.

3. The authors should include data to demonstrate that ChR2-expressing neurons in the VTA fire reliably in response to light at this duration and frequency.

4. It would be helpful to include mention of the physiological relevance of the frequency of stimulation used in vivo for VTA GABAergic/glutamatergic neurons.

5. The authors should include an information illustrating placement of optic fibers and recording electrodes for each animal.

6. Statistical table: the final column is labelled "power", but I believe the authors are reporting p-values.

## References

[B1] Amaral DG, Cowan WM (1980) Subcortical afferents to the hippocampal formation in the monkey. J Comp Neurol 189:573–591. 10.1002/cne.901890402 6769979

[B2] Amaral DG, Scharfman HE, Lavenex P (2007) The dentate gyrus: fundamental neuroanatomical organization (dentate gyrus for dummies). Prog Brain Res 163:3–22. 10.1016/S0079-6123(07)63001-5 17765709 PMC2492885

[B3] Banks MI, Li TB, Pearce RA (1998) The synaptic basis of GABA_A_, slow. J Neurosci 18:1305–1317. 9454840 10.1523/JNEUROSCI.18-04-01305.1998 PMC6792721

[B4] Beltrán JQ, Gutiérrez R (2012) Co-release of glutamate and GABA from single, identified mossy fibre giant boutons. J Physiol 590:4789–4800. 10.1113/jphysiol.2012.236372 22711957 PMC3487037

[B5] Borgius L, Restrepo CE, Leao RN, Saleh N, Kiehn O (2010) A transgenic mouse line for molecular genetic analysis of excitatory glutamatergic neurons. Mol Cell Neurosci 45:245–257. 10.1016/j.mcn.2010.06.016 20600924

[B6] Broussard JI, Yang K, Levine AT, Tsetsenis T, Jenson D, Cao F, Garcia I, Arenkiel BR, Zhou FM, De Biasi M, Dani JA (2016) Dopamine regulates aversive contextual learning and associated in vivo synaptic plasticity in the hippocampus. Cell Rep 14:1930–1939. 10.1016/j.celrep.2016.01.070 26904943 PMC4772154

[B7] Brown MTC, Tan KR, O’Connor EC, Nikonenko I, Muller D, Lüscher C (2012) Ventral tegmental area GABA projections pause accumbal cholinergic interneurons to enhance associative learning. Nature 492:452–456. 10.1038/nature11657 23178810

[B8] Castilla-Ortega E, Serrano A, Blanco E, Araos P, Suárez J, Pavón FJ, Rodríguez de Fonseca F, Santín LJ (2016) A place for the hippocampus in the cocaine addiction circuit: potential roles for adult hippocampal neurogenesis. Neurosci Biobehav Rev 66:15–32. 10.1016/j.neubiorev.2016.03.030 27118134

[B9] Chuhma N, Zhang H, Masson J, Zhuang X, Sulzer D, Hen R, Rayport S (2004) Dopamine neurons mediate a fast excitatory signal via their glutamatergic synapses. J Neurosci 24:972–981. 10.1523/JNEUROSCI.4317-03.2004 14749442 PMC6729804

[B10] Detsch O, Kochs E, Siemers M, Bromm B, Vahle-Hinz C (2002) Differential effects of isoflurane on excitatory and inhibitory synaptic inputs to thalamic neurones in vivo. Br J Anaesth 89:294–300. 12378670 10.1093/bja/aef170

[B11] Ermine CM, Wright JL, Parish CL, Stanic D, Thompson LH (2016) Combined immunohistochemical and retrograde tracing reveals little evidence of innervation of the rat dentate gyrus by midbrain dopamine neurons. Front Biol 11:246–255. 10.1007/s11515-016-1404-4

[B12] Fattorini G, Antonucci F, Menna E, Matteoli M, Conti F (2015) Co-expression of VGLUT1 and VGAT sustains glutamate and GABA co-release and is regulated by activity in cortical neurons. J Cell Sci 128:1669–1673. 10.1242/jcs.164210 25749864

[B13] Fields HL, Hjelmstad GO, Margolis EB, Nicola SM (2007) Ventral tegmental area neurons in learned appetitive behavior and positive reinforcement. Annu Rev Neurosci 30:289–316. 10.1146/annurev.neuro.30.051606.094341 17376009

[B14] Freund TF, Buzsáki G (1996) Interneurons of the hippocampus. Hippocampus 6:347–470. 10.1002/(SICI)1098-1063(1996)6:4&amp;lt;347::AID-HIPO1&amp;gt;3.0.CO;2-I 8915675

[B15] Gasbarri A, Verney C, Innocenzi R, Campana E, Pacitti C (1994) Mesolimbic dopaminergic neurons innervating the hippocampal formation in the rat: a combined retrograde tracing and immunohistochemical study. Brain Res 668:71–79. 10.1016/0006-8993(94)90512-6 7704620

[B16] Hernández-Rabaza V, Hontecillas-Prieto L, Velázquez-Sánchez C, Ferragud A, Pérez-Villaba A, Arcusa A, Barcia JA, Trejo JL, Canales JJ (2008) The hippocampal dentate gyrus is essential for generating contextual memories of fear and drug-induced reward. Neurobiol Learn Mem 90:553–559. 10.1016/j.nlm.2008.06.008 18644245

[B17] Hestrin S, Sah P, Nicoll RA (1990) Mechanisms generating the time course of dual component excitatory synaptic currents recorded in hippocampal slices. Neuron 5:247–253. 10.1016/0896-6273(90)90162-9 1976014

[B18] Hong S, Jhou TC, Smith M, Saleem KS, Hikosaka O (2011) Negative reward signals from the lateral habenula to dopamine neurons are mediated by rostromedial tegmental nucleus in primates. J Neurosci 31:11457–11471. 10.1523/JNEUROSCI.1384-11.2011 21832176 PMC3315151

[B19] Jackman SL, Beneduce BM, Drew IR, Regehr WG (2014) Achieving high-frequency optical control of synaptic transmission. J Neurosci 34:7704–7714. 10.1523/JNEUROSCI.4694-13.2014 24872574 PMC4035530

[B20] Jhou TC, Fields HL, Baxter MG, Saper CB, Holland PC (2009) The rostromedial tegmental nucleus (RMTg), a GABAergic afferent to midbrain dopamine neurons, encodes aversive stimuli and inhibits motor responses. Neuron 61:786–800. 10.1016/j.neuron.2009.02.001 19285474 PMC2841475

[B21] Kätzel D, Zemelman BV, Buetfering C, Wölfel M, Miesenböck G (2011) The columnar and laminar organization of inhibitory connections to neocortical excitatory cells. Nat Neurosci 14:100–107. 10.1038/nn.2687 21076426 PMC3011044

[B22] Kim JI, Ganesan S, Luo SX, Wu YW, Park E, Huang EJ, Chen L, Ding JB (2015) Aldehyde dehydrogenase 1a1 mediates a GABA synthesis pathway in midbrain dopaminergic neurons. Science 350:102–106. 10.1126/science.aac4690 26430123 PMC4725325

[B23] Lammel S, Steinberg EE, Földy C, Wall NR, Beier K, Luo L, Malenka RC (2015) Diversity of transgenic mouse models for selective targeting of midbrain dopamine neurons. Neuron 85:429–438. 10.1016/j.neuron.2014.12.036 25611513 PMC5037114

[B24] McNamara CG, Tejero-Cantero Á, Trouche S, Campo-Urriza N, Dupret D (2014) Dopaminergic neurons promote hippocampal reactivation and spatial memory persistence. Nat Neurosci 17:1658–1660. 10.1038/nn.3843 25326690 PMC4241115

[B25] Nair-Roberts RG, Chatelain-Badie SD, Benson E, White-Cooper H, Bolam JP, Ungless MA (2008) Stereological estimates of dopaminergic, GABAergic and glutamatergic neurons in the ventral tegmental area, substantia nigra and retrorubral field in the rat. Neuroscience 152:1024–1031. 10.1016/j.neuroscience.2008.01.046 18355970 PMC2575227

[B26] Neunuebel JP, Knierim JJ (2012) Spatial firing correlates of physiologically distinct cell types of the rat dentate gyrus. J Neurosci 32:3848–3858. 10.1523/JNEUROSCI.6038-11.2012 22423105 PMC3321836

[B27] Noh J, Seal RP, Garver JA, Edwards RH, Kandler K (2010) Glutamate co-release at GABA/glycinergic synapses is crucial for the refinement of an inhibitory map. Nat Neurosci 13:232–238. 10.1038/nn.2478 20081852 PMC2832847

[B28] Qi J, Zhang S, Wang HL, Barker DJ, Miranda-Barrientos J, Morales M (2016) VTA glutamatergic inputs to nucleus accumbens drive aversion by acting on GABAergic interneurons. Nat Neurosci 19:725–733. 27019014 10.1038/nn.4281 PMC4846550

[B29] Rocchetti J, Isingrini E, Dal Bo G, Sagheby S, Menegaux A, Tronche F, Levesque D, Moquin L, Gratton A, Wong TP, Rubinstein M, Giros B (2015) Presynaptic D2 dopamine receptors control long-term depression expression and memory processes in the temporal hippocampus. Biol Psychiatry 77:513–525. 10.1016/j.biopsych.2014.03.013 24742619

[B30] Root DH, Mejias-Aponte CA, Qi J, Morales M (2014a) Role of glutamatergic projections from ventral tegmental area to lateral habenula in aversive conditioning. J Neurosci 34:13906–13910. 10.1523/JNEUROSCI.2029-14.2014 25319687 PMC4198536

[B31] Root DH, Mejias-Aponte CA, Zhang S, Wang HL, Hoffman AF, Lupica CR, Morales M (2014b) Single rodent mesohabenular axons release glutamate and GABA. Nat Neurosci 17:1543–1551. 10.1038/nn.3823 25242304 PMC4843828

[B32] Rosen ZB, Cheung S, Siegelbaum SA (2015) Midbrain dopamine neurons bidirectionally regulate CA3-CA1 synaptic drive. Nat Neurosci 18:1763–1771. 10.1038/nn.4152 26523642 PMC11186581

[B33] Rubio-Casillas A, Fernández-Guasti A (2016) The dose makes the poison: from glutamate-mediated neurogenesis to neuronal atrophy and depression. Rev Neurosci 27:599–622. 27096778 10.1515/revneuro-2015-0066

[B34] Scatton B, Simon H, Le Moal M, Bischoff S (1980) Origin of dopaminergic innervation of the rat hippocampal formation. Neurosci Lett 18:125–131. 7052484 10.1016/0304-3940(80)90314-6

[B35] Scoville WB, Milner B (1957) Loss of recent memory after bilateral hippocampal lesions. J Neurol Neurosurg Psychiatry 20:11–21. 10.1136/jnnp.20.1.11 13406589 PMC497229

[B36] Shabel SJ, Proulx CD, Piriz J, Malinow R (2014) Mood regulation. GABA/glutamate co-release controls habenula output and is modified by antidepressant treatment. Science 345:1494–1498. 10.1126/science.1250469 25237099 PMC4305433

[B37] Stuber GD, Hnasko TS, Britt JP, Edwards RH, Bonci A (2010) Dopaminergic terminals in the nucleus accumbens but not the dorsal striatum corelease glutamate. J Neurosci 30:8229–8233. 10.1523/JNEUROSCI.1754-10.2010 20554874 PMC2918390

[B38] Stuber GD, Stamatakis AM, Kantak PA (2015) Considerations when using cre-driver rodent lines for studying ventral tegmental area circuitry. Neuron 85:439–445. 10.1016/j.neuron.2014.12.034 25611514 PMC4303766

[B39] Swanson LW (1982) The projections of the ventral tegmental area and adjacent regions: a combined fluorescent retrograde tracer and immunofluorescence study in the rat. Brain Res Bull 9:321–353. 10.1016/0361-9230(82)90145-9 6816390

[B40] Tan KR, Yvon C, Turiault M, Mirzabekov JJ, Doehner J, Labouèbe G, Deisseroth K, Tye KM, Lüscher C (2012) GABA neurons of the VTA drive conditioned place aversion. Neuron 73:1173–1183. 10.1016/j.neuron.2012.02.015 22445344 PMC6690362

[B41] Taylor SR, Badurek S, Dileone RJ, Nashmi R, Minichiello L, Picciotto MR (2014) GABAergic and glutamatergic efferents of the mouse ventral tegmental area. J Comp Neurol 522:3308–3334. 10.1002/cne.23603 24715505 PMC4107038

[B42] Tritsch NX, Ding JB, Sabatini BL (2012) Dopaminergic neurons inhibit striatal output through non-canonical release of GABA. Nature 490:262–266. 10.1038/nature11466 23034651 PMC3944587

[B43] Tritsch NX, Oh WJ, Gu C, Sabatini BL (2014) Midbrain dopamine neurons sustain inhibitory transmission using plasma membrane uptake of GABA, not synthesis. eLife 3:e01936 10.7554/eLife.01936 24843012 PMC4001323

[B44] Van Bockstaele EJ, Pickel VM (1995) GABA-containing neurons in the ventral tegmental area project to the nucleus accumbens in rat brain. Brain Res 682:215–221. 7552315 10.1016/0006-8993(95)00334-m

[B45] van Zessen R, Phillips JL, Budygin EA, Stuber GD (2012) Activation of VTA GABA neurons disrupts reward consumption. Neuron 73:1184–1194. 10.1016/j.neuron.2012.02.016 22445345 PMC3314244

